# Malaria during COVID-19 Travel Restrictions in Makkah, Saudi Arabia

**DOI:** 10.3390/tropicalmed9050112

**Published:** 2024-05-15

**Authors:** Sami Melebari, Abdul Hafiz, Kamal H. Alzabeedi, Abdullah A. Alzahrani, Yehya Almalki, Renad J. Jadkarim, Fadel Qabbani, Rowaida Bakri, Naif A. Jalal, Hutaf Mashat, Aisha Alsaadi, Ashwaq Hakim, Feras Hashim Malibari, Ahmed Alkhyami, Othman Fallatah

**Affiliations:** 1 Department of Molecular Biology, The Regional Laboratory, Ministry of Health, Makkah 21955, Saudi Arabia; samelibari@yahoo.com (S.M.); fqabbani@moh.gov.sa (F.Q.); hmmashat@moh.gov.sa (H.M.); aialsaadi@moh.gov.sa (A.A.); athakim@moh.gov.sa (A.H.); 2 Department of Microbiology and Parasitology, Faculty of Medicine, Umm Al-Qura University, Makkah 21955, Saudi Arabia; rjjadkarim@uqu.edu.sa (R.J.J.); rabakri@uqu.edu.sa (R.B.); najalal@uqu.edu.sa (N.A.J.); 3 Departments of Medical Research, Clinical Biochemistry, The Regional Laboratory, Ministry of Health, Makkah 21955, Saudi Arabia; kalzabeedi@moh.gov.sa; 4 Vector Born and Zoonotic Diseases Administration, Public Health, Ministry of Health, Makkah 21955, Saudi Arabia; abahalzahrani6@moh.gov.sa (A.A.A.); yealmalki@moh.gov.sa (Y.A.); 5 Epidemiology and Infection Control Department, Saudi German Hospital, Makkah 21955, Saudi Arabia; epi.feras@gmail.com; 6 Department of Microbiology, The Regional Laboratory, Ministry of Health, Makkah 21955, Saudi Arabia; aalkhyami@moh.gov.sa; 7 Department of Serology, The Regional Laboratory, Ministry of Health, Makkah 21955, Saudi Arabia; ofallatah@moh.gov.sa

**Keywords:** malaria, COVID-19, traveler’s malaria, severe acute respiratory syndrome

## Abstract

Malaria is a parasitic infection that may result in an acute, life-threatening illness. It is a major public health problem in the tropical world. The disease is caused by the parasites of the genus *Plasmodium* and is transmitted by female *Anopheles* mosquitoes. Saudi Arabia is in the elimination phase of malaria control. Several parts of Saudi Arabia report cases of imported malaria among travelers and visitors. The city of Makkah in Saudi Arabia has a population of about 2.3 million. Moreover, over 6 million religious visitors from different parts of the world visit Makkah annually. During the COVID-19 outbreak, travel restrictions were enforced in Makkah to contain the spread of COVID-19. We compare the total reported cases of malaria in Makkah before, during, and after COVID-19 travel restrictions in this retrospective cross-sectional study. Data on demographics, clinical data, and laboratory parameters were collected from the medical records of the Ministry of Health, Saudi Arabia. The annual malaria incidence rates in Makkah were 29.13/million people (2018), 37.82/million people (2019), 15.65/million people (2020), 12.61/million people (2021), and 48.69/million people (2022). Most of the malaria cases in Makkah were caused by *Plasmodium falciparum,* followed by *P. vivax*. Sudan, Nigeria, Yamen, Pakistan, and India are the top five countries contributing to malaria cases in Makkah. Weekly malaria case analyses revealed that COVID-19-related travel restrictions resulted in zero malaria cases in Makkah, indicating the magnitude of the travel-related malaria burden in the city.

## 1. Introduction

Malaria is the most important human parasitic infectious disease. In endemic areas, malaria has an enormous impact on public health as well as on economic development. Malaria is a vector-borne disease transmitted by the bites of infected female *Anopheles* mosquitoes. The disease is caused by *Plasmodium* parasites. There are six *Plasmodium* species that cause human malaria: *P. falciparum*, *P. vivax*, *P. ovale wallikeri, P. ovale curtusi*, *P. malariae,* and *P. knowlesi*. Malaria is widespread in the tropical areas of Africa and parts of the Americas, as well as in Asia, including the eastern Mediterranean region [1]. 

Malaria can be transmitted through more than 30 species of female *Anopheles* mosquitos [2]. Rarely, malaria can also spread through blood transfusion and organ transplant [3]. The environment, parasite species, vector population, and human host are among the various factors that determine the prevalence of a vector-borne disease [4,5]. The Arabian Peninsula is on the periphery of sub-Saharan Africa, which is a malaria-endemic region. Effective control measures have stopped local transmission in many areas of the Arabian Peninsula. However, there are still a few malaria foci that are characterized by a high incidence of drug-resistant *Plasmodium*, especially in Yemen [6,7]. 

The Kingdom of Saudi Arabia takes utmost care for the safety and health of pilgrims and religious visitors who come to Makkah. The malaria control efforts in Makkah started in the Kingdom. In 1952, DDT was used to control the vector population in Makkah. Saudi Arabia and the WHO signed a pre-eradication program plan in 1963. As a result, work on the eradication of infection vectors, extensive vector control, public health education, and training of health personnel on the management and control of malaria began quite early in the Kingdom [7]. By the late 1980s, the Makkah region was free from *An. sergentii*, which was the main malaria vector in Makkah. The other prominent malaria vector in Saudi Arabia *An. arabiensis* is not reported in Makkah; it is mostly concentrated in the south-western region of the Kingdom [8]. However, Makkah hospitals continue to get malaria cases among pilgrims and visitors that may potentially contribute to introduced malaria in the Kingdom. Introduced malaria is defined by the WHO as mosquito-borne transmission of malaria from an imported case in a geographic area where malaria does not occur regularly. Consequently, there is a need for quick improvement in the country’s malaria surveillance activities and control strategies to meet the malaria risks faced by the Kingdom [8,9]. 

In endemic areas, vector control programs, the use of insecticides, and insecticide-treated bed nets are used, and environmental interventions such as good drainage and civil infrastructure, as well as prompt diagnosis and treatment of malaria cases, help in malaria control [10]. While in places where imported malaria contributes to the most cases, prompt diagnosis and treatment are key to malaria control efforts. Therefore, information on the contribution of autochthonous vs. imported malaria in an area of interest for malaria control has high public health value. Imported malaria may result in malaria reintroduction after elimination in a geographical location [11].

Imported malaria is defined as a malaria case or infection in which the infection was acquired outside the area in which it is diagnosed. However, several variations in the definition exist. For instance, the WHO definition puts a time cap, stating that the cases should be diagnosed within 3 months of return from an endemic area, while the United States Centre for Disease Control (CDC) does not use a time limit. Within Saudi Arabia, different centers have used different definitions of imported malaria [12]. Distinguishing autochthonous vs. imported malaria in the city of Makkah is even more challenging than any other city in Saudi Arabia. Makkah has religious importance in Islam. The city of Makkah has an estimated 6 million local and international visitors for the pilgrimage of *Hajj* and *Umrah* every year. The majority of these travelers come from tropical and subtropical countries where malaria is endemic. Moreover, millions of immigrant workers come from malaria-endemic nations to seek employment in Saudi Arabia. The Muslim migrant workers living in other parts of Saudi Arabia also visit Makkah as the city has religious importance. Additionally, tens of thousands of migrant workers live in Makkah as residents. Many Saudi residents of Makkah travel on vacation and business trips to other countries and areas within Saudi Arabia where malaria is endemic [9]. The five main malaria vector species in Saudi Arabia are *Anopheles arabiensis*, *An. sergentii*, *An. stephensi*, *An. superpictus*, and *An. culicifacies*. In the Makkah region, *An. arabiensis* used to be present. However, extensive vector control efforts have eliminated the malaria vectors in large parts of Saudi Arabia [7]. A more recent study reports the absence of malaria vectors in the Makkah region [13]. In those places in Saudi Arabia where malaria vectors exist, imported malaria cases pose a major concern by spreading the infection among locals as well as other travelers. Massive travel in Makkah makes it impossible to estimate autochthonous vs. imported malaria in the city by analyzing the nationalities of the malaria cases. 

In December 2019, Wuhan, China, reported the discovery of the first case of Coronavirus disease (COVID-19). The disease is a contagious illness brought on by coronavirus 2, which causes severe acute respiratory syndrome (SARS-CoV-2) [14]. Subsequently, the disease turned into a global pandemic, forcing preventive measures such as social distancing, mandatory masks in public places, lockdowns, and curfews; there were strict travel restrictions in many parts of the world. In the city of Makkah, Saudi Arabia, travel restrictions were introduced on 23 March 2020. Moreover, the *Umrah* and *Hajj* pilgrimages were suspended in the year 2020 [15]. 

In 2021, a small number of locals were allowed in Makkah for *Umrah* and *Hajj* pilgrimages after screening for fever as well as other COVID-19 symptoms. Moreover, there were restrictions on travel by road into Makkah. People were only allowed to commute on road from 12 p.m. to 3 p.m. for about 6 months starting from 2 April 2020 [16]. While these measures were used to help limit the spread of COVID-19, these measures also resulted in a drastic reduction in the number of local and international travelers to the holy city of Makkah. For certain periods during the COVID-19 outbreak, there was unprecedented minimal human movement in and out of Makkah. 

We used these circumstances to estimate the impact of travel on malaria cases in Makkah. We collected data on malaria cases reported in Makkah before, during, and post-COVID-19-induced travel restriction periods. We analyzed malaria cases for age and nationality distribution. Our study aims to look at the trend and epidemiology of malaria cases before, during, and after COVID-19 travel restrictions in Makkah. 

## 2. Materials and Methods

Study location: Makkah is an ancient city that has central religious importance in Islam. Muslims face toward *Kaba*, which is located at *Al Masjid Al Haram* in Makkah, during prayers (*salah*). About 6 million Muslims visit Makkah every year for religious pilgrims of *Haj* and *Umrah*. The estimated population of Makkah was about 2.3 million in 2022. Makkah has an elevation of 300 m above sea level. The average annual rainfall ranges between 25 mm and 80 mm. Temperature may exceed 48 °C during summer and may touch as low as 18 °C during winter, with an average annual temperature of around 29.9 °C. Mainly two types of religious visitors come to Makkah: *Hajj* pilgrims and *Umrah* visitors. The *Hajj* is performed in the *Hijri* month of *Dhul Hijjah* while the *Umrah* is performed throughout the year. Makkah receives a bulk of *Umrah* visitors in the *Hijri* month of *Ramadan*.

Study design: This is a cross-sectional retrospective study. Data on all confirmed cases of malaria reported to the Vector-Borne and Zoonotic Diseases Administration (VBZDA) in the Department of Public Health were collected in Makkah, Saudi Arabia, from the years 2018 to 2022. The data were collected after obtaining written approval from the Ministry of Health, Saudi Arabia. The data were collected in the form of a Microsoft Excel sheet. The personal identifiers in the data were removed, and a code was given to every case to protect the identity and personal information of the patients. The data were stored in a password-protected computer. The Ministry of Health in the Kingdom of Saudi Arabia has a policy of performing thick and thin blood films on patients who have a clinical suspicion of malaria. Additionally, *Plasmodium* species are identified using molecular methods. All confirmed cases of malaria in hospitals and primary healthcare facilities must be reported to the local malaria elimination program unit within 24 h of diagnosis [17]. The malaria elimination program unit’s office then sent all the collected data to the Department of Public Health, Ministry of Health, Saudi Arabia. Weekly analyses of data are given to the Ministry of Health’s Preventive Medicine branch. Surveillance data tabulation, figure generation, and simple analysis for this descriptive epidemiological study were performed using Microsoft Excel, version 2403 and SPSS software, version 15.0 (SPSS Inc., Chicago, IL, USA).

Definitions: The following definitions were used in this study. A malaria case is defined as ‘a person with fever or history of fever with asexual malaria parasite detected in the blood during microscopic examination’. A Saudi National (citizen) is defined as a case whose identity is recorded as ‘Saudi National Identity Number’ in the data collected from VBZDA, Department of Public Health Saudi Arabia. The nationality of a foreigner is determined as his/her nationality recorded in his/her visa obtained during his relevant Makkah visit. It should be noted that some of the cases may have dual or multiple nationalities.

Diagnosis: All suspected cases were diagnosed by microscopic examination of Giemsa stained, thick and thin blood films of peripheral blood obtained from the patient. Such blood film examinations were performed up to 4 times in 24 h if the parasitemia was suspected to be very low. About 200 microscopic fields were examined before writing a negative result. Thin blood films were used for species identification. For quick diagnosis, rapid diagnostic tests were used to diagnose malaria infection and for species identification [18]. 

## 3. Results

### 3.1. Year-Wise Malaria Cases in Makkah

**Figure 1:**
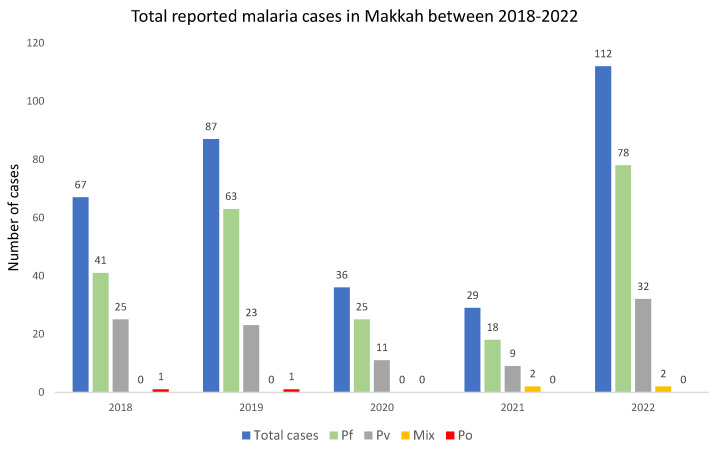
Total confirmed malaria cases in Makkah from 2018–2022. Malaria cases in Makkah declined in the years 2020 and 2021 following implementation of travel restrictions during COVID-19 pandemic. Total number of malaria cases and the cases caused by different species of malaria parasite have been displayed. Abbreviations used are *Plasmodium falciparum* (Pf), *P. vivax* (Pv), *P. ovale* (Po), and mix species infection (Mix).

Malaria cases in Makkah were studied for the years 2018–2022. The year 2021 saw the least malaria cases in Makkah at 29 cases, while the most malaria cases were reported in the year 2022 at 112 (Figure 1). Confirmed total malaria cases were 67, 87, 36, 29, and 112 in the years 2018, 2019, 2020, 2021, and 2022 respectively. The COVID-19 outbreak was at the end of 2019, and the following years, 2020 and 2021, saw the most unprecedented global lockdown and travel restrictions in human history. In the years 2020 and 2021, malaria cases in Makkah were the lowest in our study period. The most common malaria parasite detected in Makkah patients was *Plasmodium falciparum,* followed by *P. vivax*. 

### 3.2. Malaria Cases by Nationality Distribution

**Figure 2:**
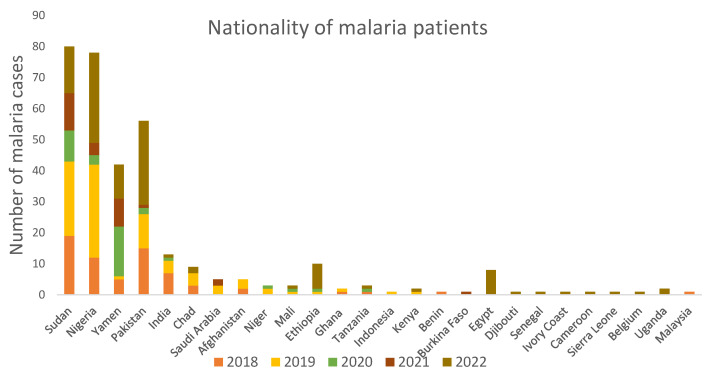
The nationality of patients diagnosed with malaria infection in Makkah in different years. Sudani, Nigerian, Yemeni, Pakistani, and Indian nationals accounted for majority of malaria cases in Makkah during the period 2018–2022.

When analyzed for the nationality of malaria-infected patients, nationals of Sudan, Nigeria, Yamen, Pakistan, and India contributed to most malaria cases in Makkah (Figure 2). Interestingly, in the year 2022, there were nine new nationalities that contributed to malaria cases in Makkah, namely Burkina Faso, Egypt, Djibouti, Senegal, Ivory Coast, Cameroon, Sierra Leone, Belgium, and Uganda. There were no malaria cases originating from these nine nationalities in the previous years studied. 

### 3.3. Malaria Cases by the Weeks

**Figure 3:**
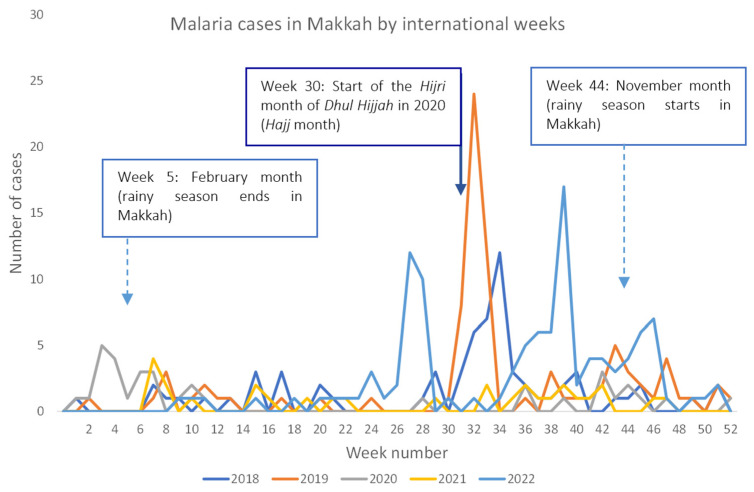
Weekly malaria cases in Makkah before, during, and after COVID-19 travel restrictions. Malaria cases tend to increase before *Hajj* due to influx of large number of long-term stayers in Makkah. *Hajj* is performed in the *Hijri* month of *Dhul Hijjah*. This corresponded to week 30 in the year 2020 (solid arrow). Hijri calendar has 354 or 355 days in a year. The month of *Dhul Hijjah* started in week 32 in year 2018 and in week 26 in year 2022. Rainy season generally starts in November (week 44) and ends in January (Week 5) in Makkah (dotted arrow signs).

We analyzed the occurrence of malaria in Makkah by week to understand the timings of malaria cases in Makkah. Makkah receives its annual rains in the winter season in the months of November to January [5]. Interestingly, malaria does not peak in rainy seasons in Makkah, suggesting that malaria cases might be independent of rainy seasons in Makkah (Figure 3). Instead, malaria cases in Makkah peak at the time of Hajj and Ramadan, the time when Makkah receives the most visitors in the year to perform *Hajj*, *Umrah,* and prayers at Al Masjid Al Haram. 

### 3.4. Malaria Cases in Makkah during Year 2020

**Figure 4:**
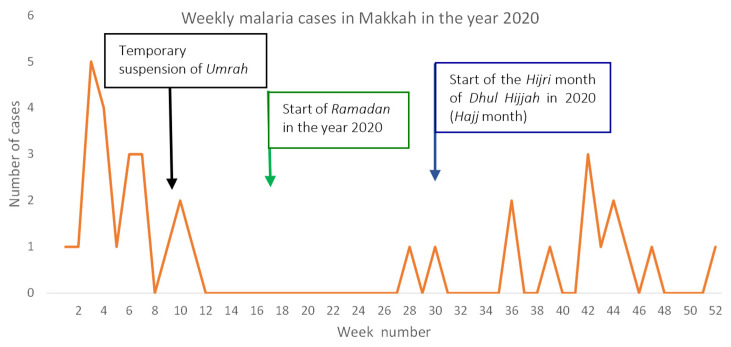
Weekly malaria cases in Makkah in 2020. Zero malaria cases in Makkah post temporary suspension of *Umrah* on 27 February 2020. Three weeks after the temporary suspension of *Umrah,* malaria cases in Makkah dropped to zero for almost 15 weeks. Green arrow indicates week 17, coinciding with the start of Ramadan in Saudi Arabia (23 April 2020), and blue arrow indicates start of *Hajj* month (22 July 2020, week 30). Black arrow indicates 27 February 2020 (week 9) when temporary suspension of *Umrah* was announced by the Government of Saudi Arabia.

Since COVID-19 lockdown greatly restricted the movement of people into Makkah at different times, we analyzed the malaria cases in Makkah during the year 2020. Interestingly, on 27 February 2020, corresponding to week 9, Saudi Arabia announced a temporary suspension of entry for Muslims wanting to perform *Umrah* pilgrimage in Makkah. We observed that there were zero malaria cases 3 weeks after this suspension of entry into Makkah. There was a long, continuous duration of 15 weeks with zero malaria cases in Makkah following this travel suspension. On 21 June 2020, Makkah was reopened for *Umrah* pilgrims, corresponding to the end of week 25. Incidentally, malaria cases reappeared in week 28 (Figure 4).

## 4. Discussion

Malaria is a major public health problem in many parts of the world. Malaria infections are generally curable; however, a small percentage of malaria cases may develop into life-threatening severe malaria. Children, pregnant women, and non-immune adults are more susceptible to severe malaria [19,20]. 

In Makkah, there are very few studies that investigated malaria transmission in the city. However, a study has reported the absence of local malaria transmission in Makkah, suggesting that all the malaria in Makkah is brought in by visitors and travelers [13]. Due to the large number of local and international travelers coming to Makkah and the complex population structure of Makkah with a large number of foreign residents [21], it is quite difficult to precisely estimate autochthonous and imported malaria in Makkah. 

*Plasmodium falciparum* is the most common malaria species detected in Makkah, followed by *P. vivax* throughout our study period. Most severe malaria cases and malaria-related deaths are attributed to *P. falciparum* infections globally. The unique cytoadherence properties of *P. falciparum*-infected erythrocytes are thought to be among its virulence properties, leading to severe disease [22,23]. Early diagnosis and treatment with effective antimalarial drugs are key to preventing falciparum infections from becoming severe disease [24,25]. 

Our study demonstrated that when travel into Makkah was comprehensively restricted during the COVID-19 pandemic, the number of reported malaria cases drastically dropped in Makkah. The Saudi Government announced the temporary suspension of *Umrah* on 27 February 2020. In the almost 3 weeks’ time since this announcement, the number of malaria cases in Makkah dropped to zero. This suggests the impact of travel on malaria in Makkah. The malaria parasite has a weeks-long incubation period in which no sign of clinical symptoms is shown after an infective bite by the mosquito. In this period, the parasite largely resides in the host liver. Malaria symptoms typically appear after the onset of the blood cycle, which starts 2–4 weeks after an infective bite. 

In endemic areas, children represent the highest burden of clinical malaria [25]. With repeated infections, adults living in endemic areas may become immune to symptomatic infections. Therefore, in a place with active malaria transmission, children are likely to represent a bulk of total malaria cases seeking medical care. Interestingly, there were very few cases of malaria that involved children younger than 5 years in Makkah throughout our study period. For instance, in the year 2018, there were 3 cases below 5 years of age: a 1-year-old, 3-year-old, and 4-year-old. In the years 2019, 2020, and 2021, there were zero cases of malaria below 5 years of age. The youngest malaria patient was an 8-year-old from Chad. In the year 2020, the youngest patient was a 12-year-old boy from Saudi Arabia. In the year 2021, the youngest patient was a 6-year-old Saudi Boy. The very low number of child malaria cases in Makkah in our study population also suggests there might be little or no local malaria transmission. 

When we analyzed the nationality of the malaria patients in our study, patients from Sudan, Nigeria, Yemen, Pakistan, and India were ranked in the top five most malaria-contributing countries in Makkah. These countries are regarded as malaria endemic, and people from these countries come to Makkah not only for *Hajj* and *Umrah* but also for work and business. It was interesting to see that in the year 2022, malaria cases were brought from new nationalities, including Burkina Faso, Egypt, Djibouti, Senegal, Ivory Coast, Cameroon, Serra Leone, Belgium, and Uganda. Since 2018, not a single malaria case in Makkah has come from nationals of these countries. There may be several reasons for this. Firstly, this may be because more people from these countries had come to Makkah for *Hajj* and *Umrah* after international travel was opened post-COVID-19 restrictions. Secondly, COVID-19 might have changed the health-seeking behavior. Sick people were more likely to be presented at the hospital to know if they had COVID-19 infection or not. And lastly, during this time, there were robust COVID-19 regulations implemented in Saudi Arabia that included putting body temperature measuring devices in public places where people may gather, such as shops, markets, etc. Therefore, people were more likely to know if they had a fever and seek medical help.

It should be noted that our data have records of the nationalities of the patients. This does not reflect the travel history. For example, the presence of a case from a Belgian national does not mean that the infection necessarily came from Belgium. The patient might have traveled to a malaria-endemic country before coming to Makkah. Belgium allows dual citizenship; the Belgian malaria patient in Makkah might be a citizen of another country as well. We recommend that while recording the travel history of malaria patients with travel history within Saudi Arabia, the names of cities should be recorded in the travel history column instead of the country. This will help researchers determine the areas within Saudi Arabia with active malaria transmission and those without malaria transmission. 

Malaria cases generally peak in Makkah when there is an influx of people in Makkah from outside. In the *Hijri* months of *Ramadan* and *Dhul*-*Hajj* (month of *Hajj*), the influx of people in Makkah is the highest. *Hajj* visitors typically stay longer in Makkah as compared to Ramadan visitors. Therefore, malaria-infected *Hajj* visitors are more likely to be diagnosed with malaria owing to the weeks-long incubation of the malaria parasite in the human body. This is seen in malaria cases, which peak around the *Hajj* months. Mosquito-transmitted diseases may peak during rainy seasons because of increased mosquito population in a location. There was a surge of dengue cases in Makkah in 2019 as compared to previous years, owing to heavy rainfall in 2019 [5]. However, in Makkah, more malaria cases were reported during the *Hijri* months of *Ramadan* and *Dhul*-*Hajj* as compared to the rainy season, suggesting the impact of travel on malaria cases. It should be noted that Makkah has relatively pleasant weather during the rainy season as compared to the rest of the year. It may be possible that in the rainy season, there are slightly more religious visitors coming to Makkah as compared to the summer. It will be interesting to determine the factors that influence malaria cases in Makkah in addition to travel during the *Hijri* months of *Ramadan* and *Dhul*-*Hajj*.

Identifying autochthonous malaria in Makkah is key for attaining malaria control and elimination within the city and the country in general. The religious importance of Makkah in Islam results in a very high influx and outflux of local and international travelers. Additionally, Makkah has a high population density. These factors can easily spread the parasite within the city and to other regions within Saudi Arabia where the vector moves. Therefore, a continuation of implementing integrated malaria preventive measures is key to preventing the resurgence and reintroduction of malaria transmission in Makkah. In addition, understating the impact of large epidemic processes such as COVID-19 on healthcare systems and the dynamics of other infectious diseases is essential for future health policy planning and preparedness [26]. Further research is needed to determine the extent of autochthonous and imported malaria as well as the presence/resurgence of malaria vectors in and around Makkah. Drug resistance in malaria parasites is a serious concern because antimalarial drugs are limited in number, and the development of novel antimalarial drugs has been slow [27,28]. Therefore, the import of drug-resistant malaria parasites in any location is a serious public health concern [29]. Since Makkah receives malaria patients of several nationalities, it is likely that global drug-resistant malaria strains may be brought to Makkah [30]. Research is needed to estimate the drug resistance in malaria strains among Makkah patients.

Limitations: We have used the data of the Ministry of Health, Saudi Arabia. The Ministry of Health has a comprehensive and very active malaria detection program in Makkah. However, during the COVID-19 outbreak, the health-seeking behavior of the local population might have changed. Therefore, the observed decrease in malaria cases might partially be due to decreased health-seeking behavior of the population due to fear that they might catch COVID-19 if they went to hospital. Additionally, the manuscript reports the nationality of the reported malaria cases in Makkah. Detailed travel history of patients may give new insights into the origin of malaria cases in Makkah. 

## 5. Conclusions

Our study demonstrates that during the most comprehensive COVID-19 travel restrictions in Makkah, there were zero malaria cases reported in Makkah. This suggests that most malaria cases in Makkah may be travel-dependent. Research studies are needed to estimate the autochthonous and imported malaria in Makkah. Outside the travel restriction period, the relatively low malaria cases in Makkah indicate the effectiveness of the malaria eradication program in Saudi Arabia as a whole. Therefore, the hospitals that cater to *Hajj* and *Umrah* pilgrims coming from endemic countries should be the main centers for tackling malaria cases in Makkah. The present surveillance and eradication measures should be consolidated. Patients who have traveled to or are coming from any of the malaria-endemic regions should be assessed for malaria infection. 

## Data Availability

Data can be obtained from the corresponding author after approval from the Ministry of Health, Saudi Arabia.
